# Improving gross motor skills of children through traditional games skills practiced along the contextual interference continuum

**DOI:** 10.3389/fpsyg.2022.986403

**Published:** 2022-11-24

**Authors:** Bahar Hussain, Jadeera Phaik Geok Cheong

**Affiliations:** ^1^Faculty of Sports and Exercise Science, Universiti Malaya, Kuala Lumpur, Malaysia; ^2^Government Degree College Shewa, Swabi, Pakistan

**Keywords:** blocked practice, random practice, practice condition, practice schedule, game-based, TGMD-2

## Abstract

Gross motor skills (GMS) are the foundation for humans reaching an optimum level of motor competence necessary to undergo normal development, maintain health, and achieve athletic excellence. Yet, there is evidence that GMS levels of children are on a decline globally. Therefore, the main purpose of this study was to investigate the effectiveness of traditional cultural games (TCG) skills, practiced according to different amounts of contextual interference (CI), on the acquisition and retention of GMS. A total of 103 Pakistani primary school children aged between 7 and 10 years were recruited for this study. Participants were randomly assigned to four practice groups with different amounts of CI: Block (B) (low interference), gradually increasing (GI) (moderate interference), random (R) (high interference), and game-based (high interference). The Test of Gross Motor Development (TGMD-2) was used to assess four tasks [overhead throw (OT), underhand throw (UT), catch (C), and throwing to a target]. The test was administered on four occasions: during pre-test, post-test, retention, and transfer. The results showed that the R group outperformed all the other groups in the post-test and the retention test. Meanwhile, in the transfer test, both R and Game-Based groups performed better than the B and GI groups. There were no differences between the R and Game-Based groups during transfer. Practicing TCG skills according to a random order was better for the acquisition and learning of GMS. The CI effect was evident, whereby high interference practice schedules were superior to low and moderate interference practice schedules.

## Introduction

Performance of motor skills is beneficial to the overall development of children, including social (Zeng et al., 2019), physiological (Cattuzzo et al., 2016), psychological (Barnett et al., 2018), and cognitive (Libertus and Hauf, 2017; Lee et al., 2020) development. However, studies have shown that the motor skill levels of children are on a decline (Brian et al., 2019, 2020), and a cause for alarm (Rechtik et al., 2019). Other studies have also shown that school children’s performance in Gross Motor Skill (GMS) assessments such as the Test of Gross Motor Development (TGMD-2) was either average or lower than average in low and middle-income countries (Bakhtiar, 2014; Jakiwa and Suppiah, 2020). This finding does not appear to be only affecting low and middle-income countries but also in high-income countries like Czech Republic (Rechtik et al., 2019) and Singapore (Mukherjee et al., 2017). In the case of Singapore, reduced motivation for physical education, physical activity and sports participation were listed as reasons for why the GMS of more than 70% of boys and girls were rated as poor (Mukherjee et al., 2017). Meanwhile, Rechtik et al. (2019) discovered that the lack of interest in movement and physical activity in Czech Republic caused more than half of the children achieving below average GMS levels. GMS are the basic skills required for movement and motor functions or manipulating activity, and involves movements of large muscles in the arms, legs, and torso. These skills are further divided into two categories of skills, Locomotor (LM) and Object Control (OC) skills (Goodway et al., 2019). To address the inferior levels of GMS, several studies have recommended intervention programs to alleviate the GMS levels of healthy and normal children. These programs include participation in sports (de Bruijn et al., 2019), GMS activities (Lee et al., 2020), integrated neuromuscular training programs (Duncan et al., 2018), unstructured programs such as free play in the pool or at the playground (Burrows et al., 2014) and more.

Besides these programmes, traditional cultural games (TCG) have also been proposed as an activity for improving motor skills. TCG are games which have developed over time, and have been descended from one generation to another (Trajkovik et al., 2018). Through these games, children individually get the opportunity to maintain their health, learn and adopt sociolinguistic skills, improve psychomotor skills, as well as other necessary elements for lifestyle endeavors (Yeniasir et al., 2017). Unlike sports, Kovačević and Opić (2014) pointed out that engaging in traditional games has no special prerequisites, nor requires infrastructure or other sports implements and appliances which may be costly. There are also no specific requirements for age, gender, or class with these games. As such, TCG would be the preferred option as a potential solution to the development of children’s GMS. In a systematic review, it was revealed that a significant number of studies conducted in China have evaluated the health benefits of traditional Chinese activities (Guo et al., 2016). Moreover, TCGs were also reported to positively influence locomotion and OC skills development compared to the usual physical activities performed during syllabus-based physical education classes (Akbari et al., 2009). With TCG, children engaged in activities that helped develop muscles, coordination, and power (Khalid, 2008), psychomotor and mental development (Bronikowska et al., 2015; Yeniasir et al., 2017), and promote physical and psychological health (Khan et al., 2019). It was also reported to be related to children’s basic needs for GMS (Charles et al., 2017). By integrating TCG into motor development programs, there is also the additional benefit of the preservation of cultural diversity, which is slowly disappearing and forgotten (Fitri et al., 2020).

The execution of TCG skills to improve GMS relies on practice. Practice is considered the more important factor to improve permanently the ability to perform motor skills (Marteniuk, 1976; Hoover et al., 1981). Practice or experience has also been argued to be responsible for general changes in the process of skill learning (Schmidt et al., 2018). For decades, researchers have been working toward finding the most effective way to conduct a skills practice session for maximum learning. One of the ways that have been found to aid the acquisition and retention of motor skills is related to the contextual interference (CI) effect. The CI effect is a well-known phenomenon found in literature surrounding motor learning referring to the interference that occurs when numerous skills, or variations of skills, are practiced in the same practice session (Shea and Morgan, 1979). Low CI occurs when one skill is consistently practiced before moving onto another skill while high CI occurs when multiple skills are practiced one after the other in a random order (Buszard et al., 2017). According to the CI effect, practicing skills under high CI conditions characterized by random practice facilitates learning. To date, many studies on CI have been conducted within various populations, for example, in children (Painter et al., 1994; Duff and Gordon, 2003; Meira and Tani, 2003; Yanci et al., 2013), adults (Goode and Magill, 1986; Wu et al., 2011; Cheong et al., 2012, 2016), persons with disabilities (Painter et al., 1994; Schweighofer et al., 2011), experts (Sharp et al., 2020), and novices (Meira and Tani, 2003; Karimiyani et al., 2013), involving a multitude of sports (Goode and Magill, 1986; Meira and Tani, 2003; Cheong et al., 2012, 2016; Karimiyani et al., 2013; Yanci et al., 2013; Sharp et al., 2020) and non-sports (Painter et al., 1994; Duff and Gordon, 2003; Schweighofer et al., 2011; Wu et al., 2011) tasks. These studies have compared the different levels of CI, such as low and high interference (Goode and Magill, 1986; Painter et al., 1994; Duff and Gordon, 2003; Meira and Tani, 2003; Schweighofer et al., 2011; Wu et al., 2011; Sharp et al., 2020), moderate interference (Cheong et al., 2012; Karimiyani et al., 2013; Yanci et al., 2013) and a game-based, open environment (Cheong et al., 2016) practice schedule. With the implementation of TCG programs in schools, there is one question that arises, which is how to increase the GMS of school children through TCG intervention. One possible solution is to apply the CI effect. That said, the CI effect has never been studied in children within a real-world environment utilizing TCG skills. Therefore, how much CI is required for the learning of TCG motor skills remains unknown. As such, the purpose of this study was to investigate the effectiveness of practicing TCG skills along the CI continuum on the acquisition and retention of GMS in primary school children. Specifically, TCG skills were practiced according to four practice schedules, either in an open or closed environment and with different amounts of CI. It was hypothesized that the moderate and high interference practice schedules would be superior for the acquisition of GMS compared to the low interference practice schedule. Additionally, it was expected that skills practiced in an open-skill environment would better facilitate learning of GMS compared to skills practiced in a closed-skill environment.

### *Pittu-Garam*, a traditional cultural game in Pakistan

*Pittu-Garam* is a popular local TCG of Khyber Pakhtunkhwa (Bashir and Zain-Ul-Wahab, 2018), the province of Pakistan. The game helps children to work as a team, and support each other at the same time (Khalid, 2008). This game involves two teams–the hitters and the seekers–in the outdoors. Players from each side use a ball to hit a *Pittu* that has a pyramid shape, made of seven stones. The hitters try to rebuild the pile while the opposing team tries to tag by hitting the member of the hitter team with the ball. The next turn will come if the *Pittu* is rebuilt or the ball hits any of the team players while rebuilding the *Pittu*. Playing *Pittu-Garam* involves running, chasing, hopping, and leaping, which are skills related to LM and throwing, rolling, catching, and hitting the target or player, which are skills related to OC.

## Materials and methods

### Participants

In total, 103 healthy, able-bodied primary school children aged 7–10 years were recruited through convenience sampling from two schools located in the Khyber Pakhtunkhwa province of Pakistan. All children had no prior experience playing the TCG (*Pittu-Garam).* The overall mean age of the children was 8.46 ± 1.21 years. A copy of the informed consent form was signed by the children’s parents and the research was carried out according to the ethical guidelines of the Universiti Malaya Research Ethics Committee (Reference number: UM. TNC2/UMREC_1047).

### Practice tasks and experimental groups

The practice tasks were based on the Pakistani TCG called *Pittu Garam*. The objective of *Pittu Garam* was to eliminate players and score points by hitting the P*ittu* (target). In this game, three motor skills are used to achieve the objective of the game, namely, overhead throw, underhand throw, and catch.

Participants were randomly assigned to four experimental groups, each with different levels of CI: (a) Block (B) (low CI), (b) gradually increasing (GI) (moderate CI), (c) random (R) (high CI in a closed environment), and (d) game-based (high CI in an open environment).

All four groups practiced four tasks using a tennis ball [overhead throw (OT), underhand throw (UT), catch (C), and overhead or underhand throw toward *Pittu* (OUTP)] in each of the 18 training sessions. The participants of B, GI, and R groups performed 20 trials of each task per session (4 × 20 = 80/session), amounting to a total of 360 trials for each task by the end of the study.

Participants of the B group practiced 20 consecutive trials of one task (e.g., OT) followed by 20 consecutive trials of the second task (e.g., UT) followed by 20 consecutive trials of the third task (e.g., C), and lastly 20 consecutive skills of the fourth task (e.g., OUTP). The order was counterbalanced across participants. The GI group practiced the first 20 trials of the four tasks in block order (e.g., OT × 5, UT × 5, C × 5, OUTP × 5), the following 40 trials in serial order (OT, UT, C, OUTP, OT, UT, C, OUTP…) and the last 20 in random order (OT, C, C, UT, OT, OUTP, UT, OUTP…). The R group practiced all 80 trials of each session’s tasks in random order, with no more than two consecutive trials of the same skill.

The GB group practiced all four tasks randomly in a game of *Pittu Garam* for 1 h and 30 min per practice session. The field used for *Pittu Garam* was 80 feet long and 40 feet wide. A centerline was drawn in the center of the field which divided the field into two equal parts. Each team in the GB group consisted of four players.

### Measurements

#### Skill performance test

**FIGURE 1:**
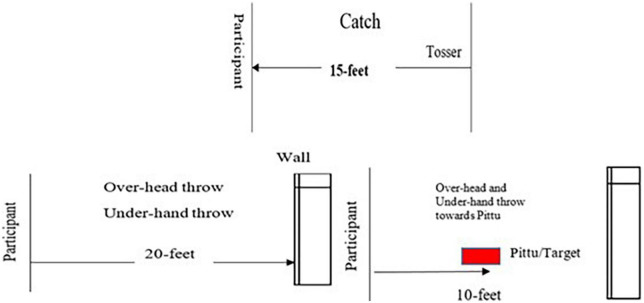
Layout of skill performance test for catch, overhead throw, underhand throw, and overhead/underhand throw towards *Pittu*.

Four tasks were assessed: OT, UT, C, and OUTP. TGMD-2 (Ulrich, 2000) was used for the measurement of OT and C while the assessment for UT was adapted from the underhand roll of the TGMD-2. The performance of the OUTP was assessed the same way as OT and UT, depending on which throw (OT or UT) was executed toward the *Pittu*. The layout for the tests is shown in Figure 1. The test comprised two trials of OT, UT, and C, respectively, and two trials each of OT and UT toward *Pittu*. The scores for these 10 trials were added to provide the total score for the Skill Performance Test. For each test, the minimum score that can be obtained is 0 and the maximum score is 38. The criteria for scoring can be found in Table 1.

**TABLE 1:** Skill performance test criteria for scoring.

1. Skill performance coding protocol for overhead throw				
**Overhead throw**	**Performance criteria**	**Trial-1**	**Trial-2**	**Score**
	
	(1) Windup is initiated with a downward movement of hand/arm	1	1	2
	(2) Rotate hip and shoulders to a point where the non-throwing side face	1	1	2
	(3) Weight is transferred by stepping with the foot opposite the throwing hand	1	1	2
	(4) Follow through beyond ball release diagonally across the body toward the non-preferred side	1	1	2

**Total marks**		**4**	**4**	**8**

**2. Skill performance coding protocol for underhand throw**				

**Underhand throw**	**Performance criteria**	**Trial-1**	**Trial-2**	**Score**
	
	(1) Take arm way forward and back to make momentum for the throw	1	1	2
	(2) Step forward with the foot opposite the preferred hand	1	1	2
	(3) Bend knees to lower body	1	1	2
	(4) Release the ball from the chest position	1	1	2

**Total marks**		**4**	**4**	**8**

**3. Skill performance coding protocol for catching a ball**				

**Catching**	**Performance criteria**	**Trial-1**	**Trial-2**	**Score**
	
	(1) Preparation phase where the hands of the child are in front of his/her body	1	1	2
	(2) Arms extend while reaching for a ball as it arrives	1	1	2
	(3) The ball is caught by hands only	1	1	2

**Total marks**		**3**	**3**	**6**

#### Game transfer test

**FIGURE 2:**
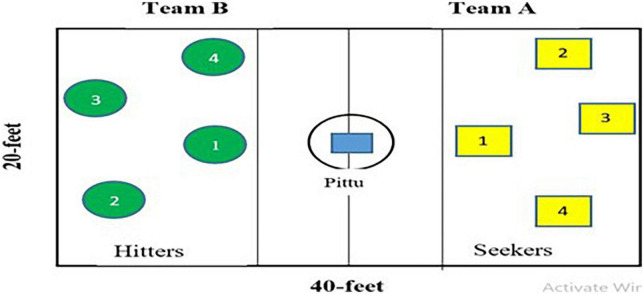
Layout of game transfer test for the game of *Pittu Garam*.

For the transfer test, three games of four versus four players were played by each of the four groups. The game was played on 40 feet long and 20 feet wide ground between two teams of the same group (see Figure 2 for layout). From the games, the first three OT, three UT, six C, and four OUTP, respectively, were assessed. The scoring followed the same criteria as the Skill Performance Test. The minimum score that can be obtained is 0 and the maximum score is 58.

### Procedures

The duration of the study was 6 weeks (three sessions/week), consisting of 18 practice sessions. One day before starting the commencement of the practice sessions, an introduction of the study was given, including a demonstration of the four tasks (OT, UT, C, and OUTP). Written cues of the skills were also distributed to the participants. A video of the skills and a game of *Pittu-Garam* were also shown to the participants. The introduction session ended with the pre-test of the Skill Performance Test. The pre-test consisted of two trials of each skill (OT, UT, C, and OUTP). All skill tests were videotaped and assessed by the principal researcher according to the TGMD-2 scoring criteria as stated in Table 1.

Participants then underwent 18 practice sessions, whereby each session comprised of the four tasks which were practiced according to the specified order based on their assigned group. All four groups received verbal indications and observational demonstrations of each skill and game at the beginning of each session. Once the participants commenced the practice, no feedback was given. After completing the last practice session, the post-test was conducted following the same format as the pre-test. The following day, the Game Transfer Test was carried out. Four weeks after the last practice session, a retention test following the same procedures as the pre-test and post-test was conducted. All matches of the game transfer test were videotaped. Participants execution of the four skills during the games were later assessed by the principal researcher according to the TGMD-2 scoring criteria as stated in Table 1.

### Statistical analysis

Data were analyzed using the Statistical Package for Social Sciences (SPSS) version 23. Four groups (B, GI, R, and game-based) × three time periods (pre-test, post-test, and retention) Split-Plot Analysis of Variance (SPANOVA) was used to analyze the Skill Performance Test. Separate ANOVAs were used to follow up significant SPANOVA analysis for each of the three time periods. For the game transfer test, scores were subjected to ANOVA. In all cases, the Bonferroni adjustment had been used for the *post-hoc* comparisons and the level of significance for all SPANOVA and ANOVA analyses was set at alpha = 0.05.

## Results

### Demographic information

The breakdown of participants according to their gender and mean age for each group can be found in Table 2.

**TABLE 2:** Distribution of participants by gender and practice group (*N* = 103).

Participant groups	Mean age (years)	Gender		Total
		Males	Females	
Block	8.46	18	07	25
Gradual increase	8.21	14	10	24
Random	8.12	21	07	28
Game based	9.07	18	08	26
Total	8.46	71	32	103

### Skill performance test

Using the SPANOVA, it was revealed that there was a significant time and group interaction, *F*(9,297) = 7.731, *p* = 0.000, $\eta_{P\,a\,r\,t\,i\,a\,l}^{2}$ = 0.637. Follow-up ANOVAs were performed at each time point. For the pre-test, there was a significant difference between groups at the start of the experiment, *F*(3, 99) = 8.959, *p* = 0.000, *$\eta_{P\,a\,r\,t\,i\,a\,l}^{2}$* = 0.214. The GB group had a significantly lower score than the other three groups. For the post-test, there was a significant difference between groups, *F*(3, 53.69) = 19.00, *p* = 0.000, $\eta_{P\,a\,r\,t\,i\,a\,l}^{2}$ = 0.321. The R group (Mean = 33.96, SD = 2.19) had significantly higher scores than the other three groups. For the retention test, there was a significant difference between groups, *F*(3, 53.140) = 37.723, *p* = 0.000, $\eta_{P\,a\,r\,t\,i\,a\,l}^{2}$ = 0.457. The R group had significantly higher scores than the GI (Mean = 28.38, SD = 4.28), B (Mean = 27.40, SD = 5.18), and GB groups (Mean = 23.23, SD = 3.88). Moreover, GI and B had significantly higher scores than GB while B and GI were not significantly different from each other. The means and standard deviations for all groups across all time points are shown in Table 3.

**TABLE 3:** Means and standard deviations of skill performance test scores for block (B), gradual increase (GI), random (R), and game-based each group at pre-, post-, and retention test.

Group	*N*	Pre-test		Post-test		Retention test	
		M	SD	M	SD	M	SD
Block	25	18.32	3.04	30.20	2.18	27.40	5.18
Gradual increase	24	19.25	3.18	30.33	2.73	28.38	4.28
Random	28	19.96	2.80	33.96	2.19	33.50	3.19
Game based	26	16.27	1.85	29.38	3.49	23.23	3.88

### Game transfer test

The ANOVA shows that there was a significant difference between groups for the game transfer test, *F*(3, 49.472) = 11.507, *p* < 0.001, $\eta_{P\,a\,r\,t\,i\,a\,l}^{2}$ = 0.270. The R group (*M* = 43.42, SD = 4.27) had a significantly higher score than GI (*M* = 36.04, SD = 7.90) and B (*M* = 33.62, SD = 8.36), whilst GB (*M* = 41.13, SD = 4.65) also had a significantly higher score than GI (*M* = 36.04, SD = 7.90) and B (*M* = 33.62, SD = 8.36). Means and standard deviations of the game transfer test for each group are shown in Table 4.

**TABLE 4:** Means and standard deviations of game transfer test for each group.

Group	*N*	*M*	SD
Block	24	33.63	8.36
Gradual increase	24	36.04	7.90
Random	24	43.42	4.27
Game-based	24	41.13	4.65

## Discussion

This study aimed to investigate the effectiveness of TCG skills, practiced according to different schedules, on the acquisition and retention of GMS in primary school children. From the skill performance test, the results showed that the random practice schedule had significantly higher scores than the B, GI, and game-based practice condition at the end of practice and retention. This finding corroborates CI literature which found that random practice, with high interference, led to superior learning compared to block practice with low interference (Battig, 1972; Shea and Morgan, 1979; Lee and Magill, 1983; Goode and Magill, 1986; Wu et al., 2011; Wright et al., 2016; Kim et al., 2018; Sharp et al., 2020). The non-consecutive repetition or unpredictability of upcoming events of the random practice encouraged deeper processing (Shea and Zimny, 1983) or enhanced forgetting (Lee et al., 1985). Specifically, the elaboration hypothesis (Shea and Zimny, 1983) argued that task interference required the learner to elaborate, compare and contrast, or relate, any memory of a previous skill that would aid in the execution of the current skill. Meanwhile, the action plan reconstruction hypothesis (Lee et al., 1985) stated that high levels of CI required the learner to reconstruct an action plan for the upcoming skill variation, and the constant reconstruction due to forgetting benefitted learning.

Surprisingly, the GI practice schedule did not outperform the R group. These results contradicted the existing literature regarding the use of moderate CI as a proposed alternative practice schedule to random practice, which causes too much interference and subsequently diminishing performance. For example, reducing the amount of interference with a gradually increasing schedule was found to benefit the learning of a dart-throwing experiment (Karimiyani et al., 2013) and a lab experiment (Porter and Beckerman, 2016). Moreover, another study found that a gradually increasing practice schedule had performed better than random practice in the learning of basketball skills (Porter and Magill, 2010). When practicing TCG skills, it was possible that the TCG, being a game, had elements of fun that could have reduced or masked the difficulty of the task, making it unnecessary to reduce the interference.

While it was hypothesized that GB would perform superior to R, these results were not supported. An earlier study (Cheong et al., 2016) reported that game-based practice, as an alternative to random practice, was better for learning field hockey skills. In that study, practicing randomly in a functional environment was suggested to promote learning by making the practice relevant to real-world settings. Moreover, there appeared to be no differences between all the groups, when skills were measured in isolation and not as part of a game. In this study, the scores of GB participants who were practicing skills while playing *Pittu Garam* were also lower than B and GI during the post-test, and no different from the B and GI during retention. This can be explained by the significantly lower pre-test scores of the GB which had much poorer skill technique at the beginning of the study. Although GB had improved over time, the improvements gained were not substantial enough to show enhanced differences from the other groups.

Aside from testing GMS in isolation and within a closed skill environment, this study also focused on the GMS executed in competitive game settings. In the Game Transfer Test, R once again performed better than B and GI, supporting the CI effect even when skills were assessed in a real game environment. In addition, GB also performed better than B and GI, supporting the proposition of game-based practice as an alternate form of random practice.

Other studies using lab tasks (Shea and Morgan, 1979; Lee and Magill, 1983; Wu et al., 2011) have also shown that random practice could produce superior performance in tests that were not similar to practice such as in transfer tests. Moreover, in field-based research, there was also support for a game-based practice schedule during transfer (Cheong et al., 2016). Similarly, another study found that random practice (tennis simulation training) in an unpredictable environment had higher transfer results for learning as compared to blocked practice (Broadbent et al., 2015). This study contributed to knowledge on CI, in that TCG skills could also demonstrate the CI effect in a novel test. Previously, transfer tests have been alluded as the “gold standard” measure of learning in an applied environment, and the results of these tests should take priority over the results of retention tests (Farrow and Buszard, 2017). According to the findings of a study, participants who practiced according to a random schedule were more resistant to changes in the environmental context (Lee and Fisher, 2019).

Lastly, on the use of TCG skills as the intervention task, previous studies have found TCGs to be beneficial within social, ethnic (Lavega et al., 2014; Anastasovski et al., 2016), and cultural (Bashir and Zain-Ul-Wahab, 2018) frameworks. Specifically, in the field of exercise science, TCG were reported to be beneficial for the health of children (Akbari et al., 2009; Abdullah et al., 2013) and adults (Guo et al., 2016). Furthermore, Andersen (2009) argued that people used to participate in TCGs for a variety of reasons, including health, leisure, and the development of endurance, stamina, and concentration in other daily tasks. Similar to practicing other types of motor and sports skills, the results of this study provided support for TCG whereby practicing TCG skills, in a random order, can help develop and improve the GMS of children aged between 7 and 10 years. The added benefit of TCG is that the participation of children in TCG may help generations to maintain culture or preserve elements of cultural traits, and to keep it safe as it keeps unites and bonds different people of the same culture (Bashir and Zain-Ul-Wahab, 2018).

The findings of this study have several practical consequences. Teachers, coaches, and other school officials must be informed of the prevalence of GMS and its influence on primary school students’ motor development. A valuable aspect is to consider TCG as a dose for the development of GMS using random practice schedules, for school children. TCG has a rich history, in that children can participate in activities that their parents and grandparents have played while growing up (Trajkovik et al., 2018). Finally, national governing bodies, parents, teachers, and school officials can make the most of GMS opportunities found in the participation of TCG.

## Conclusion

Practicing TCG skills according to random order was better for the acquisition and learning of GMS compared to a repetitive order. The CI effect was evident, whereby high interference schedules were superior to low and moderate interference practice schedules. The CI effect was also supported when practicing skills in random order in real-world settings.

## Limitations

This study used Pittu Garam, a TCG which is popular in Khyber Patunkhwa, a province of Pakistan, as an intervention to improve GMS. This game involves the use of stones, which have to be built up into a pyramid shape. Playing this game may not be appealing to all children, hence, the performance and learning may be different if this game is played by children of different cultures. Additionally, the improvements in GMS after the Pittu Garam intervention may not be generalized to all TCG from different provinces, cultures and countries. This is because of the different types of TCG worldwide, all with different ways to play, different types of apparatus, different surfaces of play due to geographical locations, different environments and so on. Every region or nation has its own traditions of music, art, fiction, dance, cultural folks, and cultural games according to the environmental issues/factors/indicators such as temperature, heat/cold, day/night shifting, rains, clouds, snow, storms, sea, mountains, and forests. It has been reported by de Barros et al. (2003) that geographical, environmental, or ecological issues and factors where children live or being brought up do have a profound or deep influence on the rate or degree of motor development in children.

## Data availability statement

The raw data supporting the conclusions of this article will be made available by the authors, without undue reservation.

## Ethics statement

The studies involving human participants were reviewed and approved by Universiti Malaya Research Ethics Committee (Reference number: UM. TNC2/UMREC_1047). Written informed consent to participate in this study was provided by the participants’ legal guardian/next of kin.

## Author contributions

BH conceptualized the research, collected and analyzed the data, and drafted the manuscript. JC conceptualized and supervised the research, interpreted the data, and reviewed the manuscript. Both authors contributed to the article and approved the submitted version.
